# The diagnostic capabilities of the combined cardiac and lung point of care ultrasound in shocked patients at the emergency department – Resourced limited country

**DOI:** 10.1016/j.ejro.2022.100446

**Published:** 2022-10-08

**Authors:** Kamonwon Ienghong, Lap Woon Cheung, Somsak Tiamkao, Vajarabhongsa Bhudhisawasdi, Korakot Apiratwarakul

**Affiliations:** a Department of Emergency Medicine, Faculty of Medicine, Khon Kaen University, Khon Kaen, Thailand; b Development of integrated Point of Care Ultrasound Used in Emergency Department, Faculty of Medicine, Khon Kaen University, Khon Kaen, Thailand; c Accident & Emergency Department, Princess Margaret Hospital, Kowloon, Hong Kong; d Emergency Medicine Unit, Li Ka Shing Faculty of Medicine, The University of Hong Kong, Pokfulam, Hong Kong; e Department of Medicine, Faculty of Medicine, Khon Kaen University, Khon Kaen, Thailand

**Keywords:** Ultrasonography, Echocardiography, Lung, Shock, Circulatory failure, Emergencies

## Abstract

**Purpose:**

Cardiac, lung, and inferior vena cava (IVC) ultrasound are commonly performed in the care of emergency patients especially patient presented with hypotension or shock. However, the literature indicated the limitation of IVC to assess shocked patients. This study aims to determine the efficacy of combined cardiac and lung ultrasound for evaluation the etiology of shock.

**Materials and Methods:**

A cross-sectional study was conducted on patient with shock at emergency department, Srinagarind Hospital, Thailand, from January to December 2021. Adult shocked patients who met the criteria were included in this study. Ultrasound and emergency department medical records were documented and analyzed as sensitivity, specificity, predictive value, negative predictive value, diagnostic accuracy, and Cohen's kappa coefficient (κ).

**Results:**

One hundred and two who met the criteria were enrolled. Combined cardiac and lung scans were found to be accurate 99.02% and 93.04% in obstructive and cardiogenic shock. In patients with obstructive shock was the almost perfect agreement, (κ) = 0.85. However, distributive, and hypovolemic shock had the low concordance with the final hospital diagnosis, (κ) = 0.37 and 0.43, respectively.

**Conclusions:**

The integration of cardiac and lung ultrasound can be effectively used to narrow differential diagnosis of shock.

## 1 Introduction

The detection of etiology of shock are critical. Some patients with shock have a clear etiology, while others are undifferentiated due to the signs and symptoms of shock might be subtle or obvious; in addition, obtaining a history can be difficult or impossible. Point of Care ultrasound (POCUS) is beneficial to provide valuable information to narrow the etiology of shock and aid the assessment of fluid status in shocked patient [1], [2].

In the last two decade, most physicians in resource limited country performed only the inferior vena cava ultrasound to evaluate patient with shock. These due to the lack of POCUS knowledge and skills to perform more complex POCUS in their patients, limited of POCUS experts, limited in the number or new function of ultrasound machines in Emergency department. Previous studies regarding the measurement of inferior vena cava (IVC) diameter as a predictor of shock [3] and the respiratory variation in IVC as the predictor of fluid responsiveness [4]. However, there are several limitations to use only IVC to assess the etiology of shock [5], [6]. According to a recent meta-analysis of the data for IVC assessment, ultrasonography does not appear to be a reliable predictor of fluid receptivity. Sensitivities and specificities were 71 % (95 %CI, 0.62–0.80) and 75 %, respectively (95 % CI, 0.64–0.85) [7].

In the absence of a reliable gold standard, assessing acute circulatory insufficiency is difficult. Focused cardiac ultrasound [8], [9], [10] has emerged as one of the most powerful techniques for clinicians to answer basic clinical issues and guide treatment in hypotensive patients. Several sonographic findings could be demonstrated include the left ventricle and right ventricle chamber sizes, left ventricle systolic function, the IVC, the presence of intramural mass, myocardial motion, and the presence of pericardial effusion. Another application is lung ultrasound [11], [12], [13] which demonstrate artifacts such as A line and B-line [14] that have been suggested to be helpful to assess volume status in hypotensive patients.

Currently, the multiorgan ultrasound system [15], [16] have been developed and applied in those patients. One of the novel diagnostic approaches employed in recent years to detect all types of shock and its causes is the Rapid Ultrasound in Shock (RUSH) protocol [17], which consists of three steps, in which POCUS was performed in multiple organs to diagnosis at the patient's bedside. Another new one is Global Ultrasound Check for the Critically ill (GUCCI) [18] which organized based on three syndromes including patient with shock. Nevertheless, these protocols may take longer time to be performed especially in the novice ultrasound practitioner.

Nowadays, there are various of dynamic measure of POCUS to assess shocked patients specially to measure patient’s cardiac output by measure left ventricular outflow tract diameter, velocity time integral. Nevertheless, that technique is complex, need to be on professional hand and limited in critical care man or anesthesiologist in resource limited country. Most clinicians at Emergency department are familiar with the gross evaluation of POCUS. Therefore, the development of POCUS curriculum in resource limited country is crucial. Currently, in Thailand, there was POCUS experts only 12 people. However, in 2018, we implemented POCUS curriculum in our emergency medicine residents. Our previous study [19] have shown that most area of POCUS examination performed by treating physician was cardiac, lung and IVC ultrasound. Moreover, competence levels of POCUS skill acquisition were evaluated and reported as satisfactory competence of image acquisition, satisfactory image interpretation skills, and good clinical integration skills.

According to this, the objective of this study aimed to propose the integrating of cardiac and lung ultrasound which currently practice by emergency physicians in resource limited country to assess the etiology of shocked patients.

## 2 Materials and methods

### 2.1 Study design and study population

This study was a single center, cross-sectional, observational analysis study obtained from shocked patients presented to the emergency department, Srinagarind hospital in Khon Kaen province, Thailand between from January 2021 to December 2021. This hospital is the medical training center and advanced tertiary care institution in northeastern Thailand, which has an average of roughly seventy thousand emergency room visit per year.

This study included adult patients who presented with shock during the study period and met the following criteria. The inclusion criteria were: (1) patients who were 18 years or older, (2) hypotension (systolic arterial pressure less than 90 mmHg, mean arterial pressure less than 65 mmHg, or a 40 mmHg reduction in systolic blood pressure from baseline), (3) hyperlactatemia (arterial lactate greater than 2 mmol per liter), and (4) patients who had only cardiac and lung ultrasound performed. Patients who satisfied one of the following criteria were excluded: (1) trauma patients, (2) cardiac arrest patients, (3) pregnant patients, and (4) patients with missing data.

### 2.2 Data collection

The data consisted of clinical and sonographic variables. Sonographic examinations were performed and recorded within 1–2 h after shocked patients visited at resuscitation room by 18 emergency medicine residents who completed 1-month POCUS training in the residency training program. The Selection of cases depended on the treating physician. Imaged were obtained and recorded by the Mindray M9 (Mindray, Shenzhen, China) which was the regular ultrasound machine used in emergency room. The instrument equipped with a curved array probe (1.4–5.1 MHz), phased array probe (1.1–4.4 MHz), and linear probe (3–13 MHz) and allowed users to acquire two-dimensional imaging using the M-mode, B-mode, and Color Doppler modes. Four standard views of cardiac ultrasound including parasternal long axis view, parasternal short axis view, subcostal view, and apical four chamber view were recorded to mainly assess right ventricle chamber sizes, left ventricle systolic function, aortic root size, the presence of pericardial effusion, and other cardiac ultrasound findings: for example, intramural mass, the presence of left ventricle D shape, Tricuspid annular plane systolic excursion (TAPSE), or valvular abnormality. Lung ultrasound was performed on eight-zone scanning area of the anterolateral according to previous study [20] to evaluate lung sliding, A-lines, B-lines, consolidation, and pleural effusion. Each ultrasound video clips were recorded at least 6 s. Patient characteristics, diagnosis given by using cardiac and lung sonographic findings (POCUS diagnosis), and final hospital diagnosis were all acquired from electronic medical record. The diagnosis was declared as four categories of shock including (1) cardiogenic shock, (2) hypovolemic shock, (3) obstructive shock, and (4) distributive shock. The results of the ultrasound examination and clinical data were documented in a standardized format by two independent investigators who was blinded to the treatment team. After the patient was discharged or died, the clinical and ultrasonic characteristics were put into the database. The duplicate data entry was completed. If the data did not match, senior investigator who had more than ten years of experience with ultrasounds pro-vided the final decision of data collection.

### 2.3 Study size

The sample size was calculated based on sample size estimation in diagnostic test studies of biomedical informatics formula [21]. The estimate for proportion of study population was made using data from a previously published study [22]. This study set standard normal deviate at 1.96, sensitivity at 0.923, and degree of accuracy at 0.10. A sample size of 102 people was determined to be necessary.

### 2.4 Statistical analysis

The data was input into Microsoft Excel and analyzed with IBM SPSS for Windows version 27.0, which is licensed to Khon Kaen University (SPSS Inc., Chicago, IL, USA). Un-less otherwise stated, continuous variables are reported as mean and standard deviation, and categorical variables are presented as number (n) or frequency (percent). The sensitivity, specificity, positive predictive value, negative predictive value, and accuracy of diagnosis were calculated. Cohen's kappa coefficient (κ) was used to compare the agreement between POCUS diagnosis and the final hospital diagnosis. The interpretation of the k coefficient values was followed in the six categories [22].

### 2.5 Ethical consideration

The Khon Kaen University Ethics Committee for Human Research approved this project (HE651088). Patients' informed agreement was not required because patient anonymity was protected by using a unique study number.

## 3 Results

The study was conducted over a period of 12 months. A total of 130 patients who met the criteria were enrolled. Data were collected for 102 patients, with 28 had incomplete data. Male patients were represented as 61 % in this study. The mean age was 52.8 ± 20.0 years. The mean systolic and diastolic blood pressures were 74.8 ± 12.3 and 50.2 ± 10.28, respectively. The most common diagnosis in the POCUS diagnosis was hypovolemic shock (50.98 %). The majority of patients (45.09 %) were diagnosed with distributive shock in the final hospital diagnostic, as shown in Table 1.

**Table 1:** Final hospital diagnosis and POCUS diagnosis in 102 patients.

Category of shock	POCUS diagnosis (%)	Final hospital diagnosis (%)
Cardiogenic	12 (11.76)	15 (14.70)
Hypovolemic	52 (50.98)	37 (36.27)
Obstructive	3 (2.94)	4 (3.92)
Distributive	35 (34.31)	46 (45.09)

**Fig. 1:**
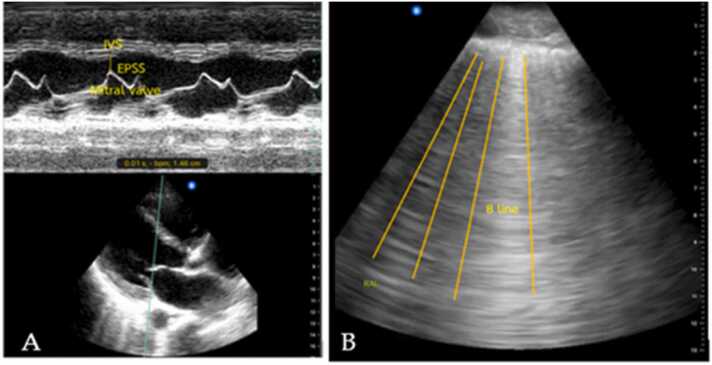
Representative of ultrasound image findings for cardiogenic shock; decrease left ventricle contraction (A) and multiple B-lines (B).

**Fig. 2:**
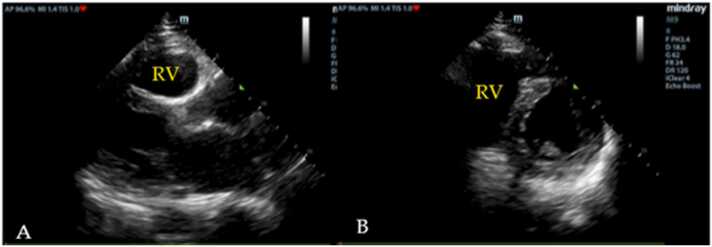
Representative of ultrasound image findings for obstructive shock; right ventricle (RV) dilatation in parasternal long axis view (A) and right ventricle dilatation in parasternal short axis view (B).

**Fig. 3:**
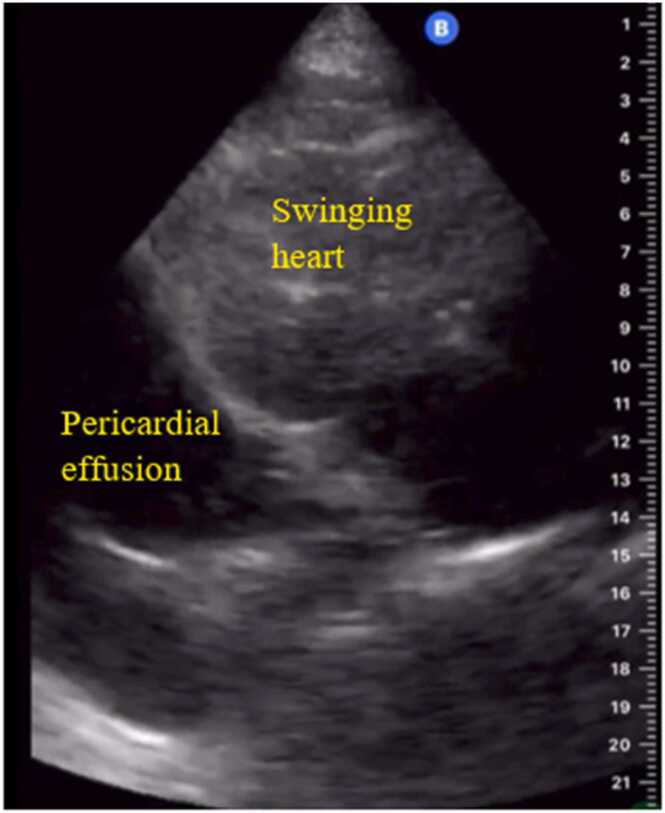
Representative of ultrasound image findings for obstructive shock; large pericardial effusion with swinging heart.

**Fig. 4:**
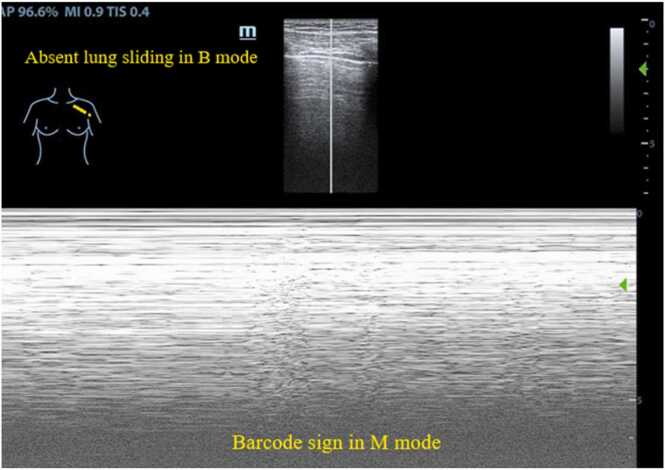
Representative of ultrasound image findings for obstructive shock; Absent lung sliding in B mode and Barcode sign in M mode.

In terms of the cardiac and lung sonographic pattern of hemodynamics in shock patients (Table 2), reduced left ventricle contractility (100 %), followed by multiple B-lines (80 %) were found in cardiogenic shock (Fig. 1). In hypovolemic shock, there are no pathological changes in the ultrasound image of the lungs; however, the hyperdynamic left ventricle found in 81.1 %. Sonographic findings which demonstrated in obstructive shock were right ventricle dilatation (50 %) (Fig. 2), followed by pericardial effusion with tamponade (25 %) (Fig. 3), decrease Tricuspid annular plane systolic excursion (TAPSE) (25 %), and absent lung sliding (25 %) (Fig. 4). Our study discovered diverse levels of left ventricle contractility in distributive shock, including hyper-dynamic left ventricle (76.09 %), faired left ventricle contractility (13.04 %), and reduced left ventricle contractility (10.87 %). From lung ultrasound signs in distributive shock, 11 (23.91 %) out of 46 revealed signs of pulmonary consolidation, including an air bronchogram in 8 cases (17.39 %).

**Table 2:** The list of sonographic findings detected from ultrasound in patients diagnosed from final hospital diagnosis divided from each type of shock.

Final hospital diagnosis	Signs from cardiac ultrasound		Signs from lung ultrasound	
Cardiogenic shock (n = 15) (%)	Reduced left ventricle contractility	15 (100)	B-line	12 (80)
	Pericardial effusion	1 (6.67)	Pleural effusion	4 (26.67)
Hypovolemic shock (n = 37) (%)	Hyperdynamic left ventricle	30 (81.1)	A-line	37 (100)
	Small left ventricle chamber	7 (0.19)		
Obstructive shock (n = 4) (%)	Pericardial effusion with tamponade	1 (25)	Absent lung sliding	1 (25)
	Right ventricle dilatation	2 (50)		
	Decrease TAPSE	1 (25)		
Distributive shock (n = 46) (%)	Hyperdynamic left ventricle	35 (76.09)	A-line	41 (89.13)
	Reduced left ventricle contractility	5 (10.87)	B-line	5 (10.87)
	Faired left ventricle contractility	6 (13.04)	Pleural effusion	2 (4.35)
			Consolidation	11 (23.91)
			Air bronchogram	8 (17.39)

Among four type of shock, POCUS diagnosis for patient diagnosed with hypovolemic shock had greatest sensitivity (81.08 %). It was observed that the highest specificity (100 %) and the accuracy of POCUS diagnosis (99.02%) found in patient diagnosed with obstructive shock. The sonographic obstructive (κ = 0.85) and cardiogenic (κ = 0.70) pattern was considered concordant to the final hospital diagnosis when this latter was hypovolemic and distributive (Table 3).

**Table 3:** Reliability indices and agreement of POCUS diagnosis (combined clinical and POCUS examination) vs Final hospital diagnosis.

Reliability indices	Cardiogenic shock (n = 15)	Hypovolemic shock (n = 37)	Obstructive shock (n = 4)	Distributive shock (n = 46)
Sensitivity (%)	66.67	81.08	75.00	54.35
Specificity (%)	97.70	66.15	100.00	82.14
PPV (%)	83.33	57.69	100.00	71.42
NPV (%)	94.44	86.00	98.99	68.66
Accuracy	93.14	71.57	99.02	69.61
Cohen’s kappa coefficient (κ)	0.70	0.43	0.85	0.37

## 4 Discussion

Shock could be difficult to manage if clinician can’t detect the etiology of shock. According to this, POCUS provided the protocol to solve this problem. The most popular one is RUSH protocol which exhibited highly pooled sensitivity 87 % and specificity 98 % [17]. However, the multiple organs must be performed to complete that examination which could be take a longer time. Moreover, some organs may be difficult for novice sonographer to perform, for example, deep venous scanning.

This study conducted in 102 shocked patients demonstrated two vital organs scanning included cardiac and lung could determine etiology of undifferentiated shock patients which was consistent with previous ultrasound protocols that have some part of cardiac and lung ultrasound in those protocols [23], [24], [25]. Our study illustrated combined cardiac and lung ultrasound was effectively at least in substantial and almost perfect agreement with cardiogenic and obstructive shock in final hospital diagnosis, respectively. This result was consistent with another study [26] which shown the Kappa's correlation coefficient for comparing the RUSH protocol.

Among four type of shock, clinical diagnosis of obstructive shock was difficult. Our study demonstrated cardiac and lung ultrasound had highest specificity (100 %) and accuracy (99.02 %) to identify this type of shock. Cardiac and lung ultrasound showed right ventricle dilatation in patient diagnosed with pulmonary embolism, pericardial effusion with tamponade and absent lung sliding in patient diagnosed with pneumothorax. These sonographic findings which was similar to previous studies [27], [28], [29] can easily and immediately perform to diagnose this type of shock which was time sensitive disease.

Most type of shock in this study was distributive shock (45.09 %) which was similar to previous study [30]. Cardiac ultrasound can be a useful tool in the septic patient because it enables for early detection of sepsis-related cardiac dysfunction which our study identified various level of cardiac contractility in this group. Another study [31] demonstrated cardiac ultrasound may identify an unanticipated critical finding in sepsis patient. Moreover, B-line artifacts finding appeared to represent the left ventricular function and volume status of the patient [32], [33]. However, the accuracy of POCUS diagnosed in distributive shocked patient was only 69.61 % which the agreement of with the final hospital diagnosis was fair. This was contrast to previous study [22].

Our study noticed the important findings which can be rapidly aid treating physician to identify type of shock in most patient that was (1) reduced left ventricle contractility and B line were found in cardiogenic shock, (2) All hypovolemic patients had A-line, (3) patient with distributive shock can have variety of left ventricle contractility level, and (4) lung ultrasound can identify pulmonary infection which may be found in distributive shock.

The study's limitations were (1) This was the single center study, which may have a different perspective on the studied population than other organizations. As a result, data should be gathered from a variety of research organizations, (2) this study was observational study, (3) this study did not compare the reliability index of using IVC scanning in shocked patient, (4) this study did not compare other diagnostic methods of POCUS in shocked patient due to it did not practically perform in our ER, (5) The expertize and skill of the ultrasonographic operator was not evaluated in this study, and 6) our research did not demonstrate the correlation between POCUS examination and changes in patient treatment and patient outcomes.

The result of this study which used POCUS diagnosis demonstrated lower number of reliability indices than previous study [22], [34]. This implies that, first, while clinical assessment or POCUS alone are both inaccurate in accurately identifying a patient with unexplained shock, using POCUS in conjunction with clinical evaluation increases diagnostic accuracy in the ED and can help guide appropriate therapy. Secondly, multiple body regions of scanning may increase reliability indices since people are complicated and generally have multiple comorbidities.

## 5 Conclusions

Integrated cardiac and lung POCUS obtained in the emergency department demonstrated fair to almost perfect agree with a post hoc clinical analysis of the etiology of shock. This study suggests that this ultrasound approach performed in these patient group was useful for identify etiology of shock and used in routine practice in emergency department in resource limited country.

## Funding

This research was supported by Faculty of Medicine, Khon Kaen University, Thailand (Grant number IN65225).

## CRediT authorship contribution statement

**Kamonwon Ienghong**: Conceptualization, Methodology, Validation, Formal analysis, Investigation, Data Curation, Writing - Original Draft, Writing - Review & Editing, Project administration, Funding acquisition, **Lap Woon Cheung**: Conceptualization, Validation, Formal analysis, Resources, Writing - Original Draft, Supervision, **Somsak Tiamkao**: Methodology, Investigation, Resources, Visualization, Supervision, **Vajarabhongsa Bhudhisawasdi**: Formal analysis, Investigation, Resources, Visualization, Supervision, **Korakot Apiratwarakul**: Conceptualization, Methodology, Validation, Formal analysis, Investigation, Data Curation, Writing - Original Draft, Writing - Review & Editing, Project administration.

## Ethical statement

Institutional Review Board Statement: The study was conducted according to the guidelines of the Declaration of Helsinki, and approved by Ethics Committee of Khon Kaen University (HE651088).

## Informed Consent Statement

Informed consent from the patients was waived since patient confidentiality protection had been guaranteed as patients were not identified by name, but rather by a unique study number.

## Declaration of Competing Interest

The authors declare that they have no known competing financial interests or personal relationships that could have appeared to influence the work reported in this paper.
